# Rituximab depletion of intrahepatic B cells to control refractory hepatic autoimmune overlap syndrome

**DOI:** 10.1093/qjmed/hcz161

**Published:** 2019-06-27

**Authors:** G D Appanna, T P I Pembroke, K L Miners, D A Price, A M Gallimore, K Ladell, A J Godkin

**Affiliations:** 1 Division of Infection and Immunity, Cardiff University School of Medicine; 2 Department of Gastroenterology and Hepatology, University Hospital of Wales, Heath Park, Cardiff, UK

**Learning points for clinicians**

- Hepatic autoimmune overlap syndromes should be considered in patients with evidence of more than one autoimmune hepatic disease.
- Disease resistant to standard immunosuppression and treatment of cholestasis can progress rapidly to cirrhosis and associated complications.
- Specialist input and treatment with novel therapies, such as B-cell depleting agents, present an opportunity to achieve disease remission in refractory cases.

##  Background

Hepatic overlap syndromes often present with features of autoimmune hepatitis (AIH) and primary biliary cholangitis (PBC). Treatment with antiproliferative agents or steroids can prevent disease progression, but immunosuppression is generally less successful in the presence of marked cholestasis. Rituximab has been used to deplete B cells in a small number of patients with AIH.^1^ However, the effects of rituximab in refractory hepatic overlap syndrome remain unknown, both clinically and immunologically.

## Case presentation

Two sisters with biochemical and histological evidence of AIH/PBC overlap syndrome were recruited from the hepatology clinic at the University Hospital of Wales (Table 1). Patient 1 failed to achieve biochemical remission on azathioprine, prednisolone and ursodeoxycholic acid (UDCA). Patient 2 did not tolerate azathioprine, prednisolone or UDCA, and was treated instead with budesonide and mycophenolate mofetil, which failed to prevent further clinical deterioration. A second liver biopsy demonstrated overlap with progressive fibrosis to early cirrhosis in Patient 2. Rituximab salvage therapy was commenced in both patients, alongside ongoing immunosuppression. Each patient received a 1 g infusion of rituximab at weeks 0 and 2. Patient 1 also received a 1 g infusion of rituximab at weeks 52 and 54. Hepatic fine needle aspiration (FNA) was performed using a 22G spinal needle as described previously.^2^ Peripheral blood and FNA cells were collected prior to rituximab infusion at weeks 0 and 2. Patient 1 underwent further sampling at weeks 26, 52 and 54.

**Table 1:** Clinical, biochemical and histological characteristics of each patient at diagnosis and before salvage therapy with rituximab

	Patient 1	Patient 2
Age	58	62
HLA typing	A2, A30, B15 (B62), B18 DR3, DQ2, DQ3, DQB103 (DQ8)	A24, B27, B35 DR4, DR7, DQ2, DQ8
Other autoimmune conditions	Type 1 diabetes mellitus Hypothyroidism	Celiac disease
Antibodies	ANA^–^ SMA^+^ (1:100) AMA^+^ (1:1600)	ANA^–^ SMA^+^ (1:25) AMA^+^ (1:1600)
ALP (30–150 IU/L)	634	403
ALT (<50 IU/L)	105	204
IgG (6–16 g/L)	19.8	19.2
IgM (0.5–2 g/L)	4.98	8.33
Liver biopsy	Interface hepatitis with no fibrosis Granulomas present with biliary changes	Interface hepatitis with bridging fibrosis Granulomas present with biliary changes
Established immunosuppression (total daily dose)	Azathioprine 100 mg Prednisolone 10 mg UDCA 1500 mg	Budesonide 3 mg Mycophenolate mofetil 2 g
IAIHG score	8	7
Biochemistry pre-rituximab therapy		
ALP (30–150 IU/L)	339	243
ALT (<50 IU/L)	54	139
IgG (6–16 g/L)	17.9	12.3
IgM (0.5–2 g/L)	4.06	2.87

Reference values are shown in parentheses for ALP, ALT, IgG and IgM. Titers are shown in parentheses for autoantibodies.

HLA, human leukocyte antigen; ANA, antineutrophil antibody; SMA, smooth muscle antibody; AMA, antimitochondrial antibody; IAIHG, International Autoimmune Hepatitis Group.

**Figure 1:**
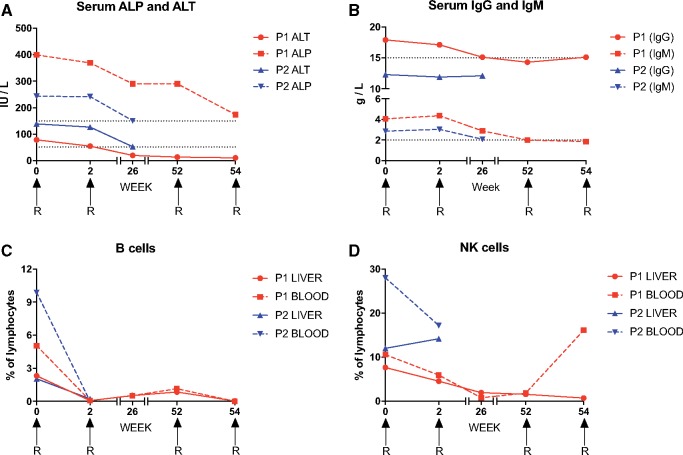

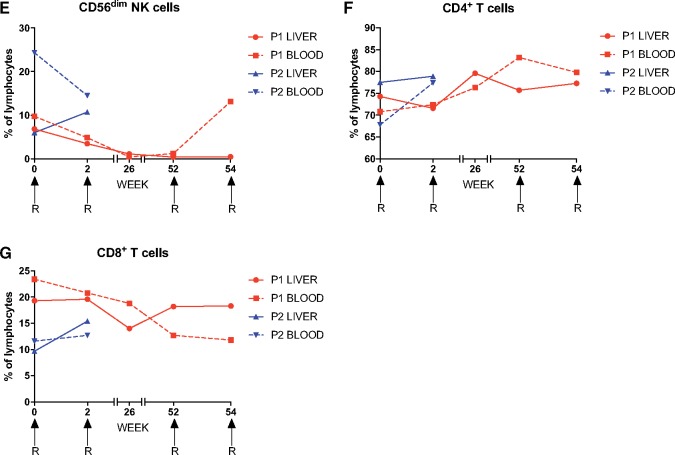
The response to rituximab infusion (denoted by R) in two sisters with refractory hepatic overlap syndrome. (**A**) Peripheral blood ALP and ALT levels. Dotted lines indicate the upper limits of normal. (**B**) Serum IgG and IgM titers. Dotted lines indicate the upper limits of normal. (**C**–**G**) Proportions of intrahepatic and peripheral blood lymphocyte subsets. (C) B cells. (D) NK cells. (E) CD56^dim^ NK cells. (F) CD4^+^ T cells. (G) CD8^+^ T cells.

Clinical improvement was apparent in both patients, who reported less fatigue. Serum concentrations of alanine transaminase (ALT), alkaline phosphatase (ALP) and immunoglobulins (IgG and IgM) also fell progressively after treatment (Figure 1A and B). Lymphocyte subsets were monitored in parallel using polychromatic flow cytometry. As expected, rituximab therapy induced a rapid loss of peripheral and intrahepatic B cells, defined by surface expression of CD19. In Patient 1, peripheral and intrahepatic B cells emerged again at weeks 26 and 52, but waned to undetectable levels after further doses of rituximab at weeks 52 and 54 (Figure 1C). Intrahepatic NK cells were also depleted in Patient 1 (Figure 1D and E). There were no substantial changes in the phenotypic attributes (data not shown) or relative frequencies of CD4^+^ or CD8^+^ T cells after rituximab therapy (Figure 1F and G).

## Discussion

This is the first report of effective rituximab salvage therapy for refractory hepatic overlap syndrome. Our data also show that rituximab can deplete intrahepatic as well as peripheral B cells, which are thought to perpetuate the disease by presenting autoantigens to pathogenic T cells.^3^

Rituximab depletes B cells via antibody-dependent cellular cytotoxicity, which is mediated *in vitro* by CD56^dim^ NK cells.^4^ In Patient 1, rituximab therapy coincided with a profound loss of CD56^dim^ NK cells, potentially reflecting the ongoing use of azathioprine.^5^ Recrudescent B cells were nonetheless eradicated by further doses of rituximab, suggesting the existence of additional depletion mechanisms in the absence of CD56^dim^ NK cells.

Hepatic overlap syndromes are difficult to manage clinically, especially if standard immunosuppressive regimens are ineffective. This report shows that rituximab can arrest refractory disease. A registry of patients with refractory hepatic autoimmune and overlap syndromes would facilitate a more systematic evaluation of long-term outcomes and inform future randomized clinical trials designed to assess the efficacy of rituximab therapy.

## Funding

TPIP is supported by an Academy of Medical Sciences Starter Grant for Clinical Lecturers and the Ser Cymru II Programme, which is funded by Cardiff University and the European Regional Development Fund via the Welsh government. DAP is a Wellcome Trust Senior Investigator.

*Conflict of interest*: None declared.
